# Influence of rhythmic-movement activity intervention on hot executive function of 5- to 6-year-old children

**DOI:** 10.3389/fpsyg.2024.1291353

**Published:** 2024-03-01

**Authors:** Suxia Wang, Anning Yang, Xuefeng Wei, Ruohan Qian, Ying Chen, WenJing Bi, Bisheng Hu, Cheng Wen

**Affiliations:** ^1^College of Elementary Education, Ludong University, Yantai, China; ^2^Zhejiang Normal University, Hangzhou City, Zhejiang Province, China; ^3^Zhejiang Beima Education and Technology Co., LTD. Enterprise Postdoctoral Workstation, Hangzhou City, Zhejiang Province, China; ^4^Zhejiang Beima Education and Technology Co., LTD., Hangzhou City, Zhejiang Province, China

**Keywords:** rhythmic activity training, hot executive function, intervention program, affective decision making, gratification delay

## Abstract

Hot Executive Function (hot EF) refers to cognitive process involved in high emotion or motivation, and the operation of this function is related to the activities of the ventromedial prefrontal lobe and orbitofrontal lobe. Meanwhile, rhythmic-movement activity is a musical activity in which one expresses and feels music with one’s own body movements which involves cognitive abilities such as adjusting and understanding emotions among children. To explore how rhythmic-movement activity with rewards influences the development of hot EF in children of 5–6 years old, the organization principles of rhythmic-movement activity with rewards intervention on hot EF were designed, and 62 children of 5–6 years old in a kindergarten in Yantai of China were selected as research participants (*M* = 5.80 years old, SD = 0.37 years old) for pre-test and post-test experimental design. The experimental group received rhythmic-movement activity with rewards three times a week for 6 weeks, while the control group did not. The gift delay task and the children’s gambling task were used to measure two sub-components of hot EF before and after the intervention, and the results show that rhythmic-movement activity with rewards has a significant effect on gratification delay and affective decision-making ability of children. Finally, the effects and enlightenment of rhythmic-movement activity with rewards on hot EF are discussed.

## Introduction

Primary school is an important turning point for children to receive formal education, during which period new learning content and classroom rules will bring challenges to children. Some children are unable to cope with these challenges and develop adjustment problems. It is usually characterized by restlessness, incessant activity, difficulty in waiting, interference in the activities of others, etc., which is specifically reflected in behaviors and emotional problems such as uncontrollable behavior, excessive activity, pursuit of timely rewards, and emotional dysregulation (Çökük and Kozikoglu, 2020; Shakehnia et al., 2021). These manifestations of impulsivity, risk-taking, and emotional disturbance may be closely related to emotion-related hot executive function (hot EF) (Prencipe et al., 2011). Low level of hot EF not only influences their immediate lives, but also have negative impact on children’s future development, such as academic and social difficulties, increased criminal activity, use of public aids, and drug abuse (Racz et al., 2013; Corcoran et al., 2018). From this perspective, improving the level of hot EF of children may be the key to solving the adaptation problem.

Hot EF refers to cognitive process involved in high emotion or motivation, and the operation of this function is related to the activities of the ventromedial prefrontal lobe and orbitofrontal lobe (Zelazo and Cunningham, 2007; Logue and Gould, 2014). The essence of hot EF is linking cognitive process with emotion, adjusting cognitive conflicts related to emotion, and making choices and actions that are in line with long-term interests and facilitate people to make good social adaptations (Rueda et al., 2016). The key stage of hot EF development is preschool, with 5 years old being the key period and turning point (Traverso et al., 2015; Veer et al., 2017). Children’s daily life is closely related to the development of hot EF. For example, children often face cognitive dilemmas related to emotions and desires, such as (i) whether to do homework before or after playing and (ii) whether a delicious snack is healthy or not. When making such decisions, children must reconcile specific emotions, expectations, and rules in the brain, manage emotions among competing choices, make plans or choices in line with long-term interests, and adjust their impulse to get instant satisfaction (Rueda and Paz-Alonzo, 2013). Hot EF can help children better adjust cognitive factors such as emotion, motivation, desire, personal social behavior norms, rewards, and win–lose emotions, affective decision making ability, and reasoning, empathy, self-awareness, etc. (Zelazo and Müeller, 2002; Fatwikiningsih, 2016). to help young children make choices and actions in line with their future development.

Some studies indicate that the core components of hot EF include gratification delay (GD) and affective decision making (ADM) (Zelazo and Carlson, 2012; Antonini et al., 2015; Crone and Steinbeis, 2017; O'Toole et al., 2019). GD refers to the choice orientation of voluntarily giving up immediate gratification for more-valuable long-term goals, and the self-control ability demonstrated in the waiting process (Yu et al., 2016; Moriguchi et al., 2018). For example, to achieve academic success, one must give up things that can produce short-term happiness (e.g., chasing dramas and games) and do things that are slow but rewarding (e.g., reading, exercising, and finishing homework). During this period, individuals need strong self-control to resist temptations and deal with setbacks. The tools for measuring GD in studies include the marshmallow experiment (Mischel, 2014), the post-picture finding task (Simpson and Riggs, 2007), the candy delay task, the gift wrapping task (Kochanska et al., 1996), and the gift delay task (GDT). Research has shown that GD appears in early childhood (Yanaoka et al., 2022), and GD in early childhood can predict social communication ability and academic achievement in adolescence, as well as weight, health, and career achievement in adulthood (Schlam et al., 2013). ADM is the ability to make choices that seek advantages and avoid disadvantages when individuals face at least two different value contradictions that cause emotional conflicts (Bracha and Brown, 2012; Charpentier et al., 2016). There are four explanations for the development of ADM ability: (i) the development of somatosensory marker ability. This theory implies that the brain influences the decision-making process through physical sensations and affective responses. In the process of decision-making, people will have physical feelings related to a specific situation. These physical feelings can be physiological changes (such as increased heart rate, increased breathing) or physical manifestations (such as facial expressions, physical movements), which provide a concrete and perceptible experience for decision-making, and this experience is crucial to the development of decision-making.; (ii) to stimulate the development of flexible representation ability of enhancement value; (iii) the development of rule reasoning and rule integration ability; (iv) focusing on the future development of decision-making ability, this being the core reason for the development of ADM ability (Zeng, 2010). The main purpose of ADM ability is to focus on the future and make choices and decisions that are beneficial to oneself or society in present activities, and it is a very important example of social adaptability (Lerner et al., 2015). When a person’s ADM ability is poor (e.g., the abuse of drugs), it leads to poor emotional regulation ability and makes it easy to do dangerous things (Volkow et al., 2010; Martin and Delgado, 2011). In addition, people with defective ADM are prone to behavioral problems such as alcoholism, smoking, addiction, etc. and are easily tempted by emotions and rewards to make choices that have negative impacts on the future. Research shows that ADM ability plays an important role in children’s cognitive ability and emotional development, and it is an important part of higher-order thinking, which runs through all conscious activities of human beings (Bandyopadhyay et al., 2013). The tools for measuring ADM ability include the emotion regulation scale (Martins et al., 2020) and the children’s gambling task (ChGT) (Kerr and Zelazo, 2004). For example, one research used the ChGT to measure how hot EF affected physical aggression in children between the ages of five and six, and this method is more suitable for testing younger children (O'Toole et al., 2019).

A way of stimulating the development of hot EF is to use sport-based interventions. Studies have shown that sport intervention can not only promote physical development and reduce the probability of obesity (Mandolesi et al., 2018) but also increase the neuronal connection between the cerebellum and frontal cortex, making attention more concentrated and promoting the development of cognitive ability (Budde et al., 2008). Physical sport is also closely related to the development of emotion, decision-making, communication skills, and social skills (Sohrabi, 2019). Study discussed the effects of different intensities of sport on children’s EF behavior. The experimental group and the control group, respectively, conducted low-intensity or moderate-intensity sport for 8 weeks, twice a week and for 35 min each time. The intervention course was aimed at helping children to coordinate sports in an interesting and pleasant way instead of focusing on competition or skill improvement, and it was found that moderate-intensity sport is best for promoting children’s cognitive and physical development (Chang et al., 2013). Compared with the promotion of long-term chronic exercise on the development of brain function, a single acute exercise also has a transient impact on growth factors such as brain-derived neurotrophic factor. Studies found that these growth factors closely related to cognitive ability increased after exercise (Diamond, 2006). Physical movement is also strongly linked to the development of emotions, decision-making, communication skills, and social skills (Sohrabi, 2019). One study reviewed the influence of Quadrato Motor Training sports training on hot EF. In motor inhibition training, practitioners were required to alternate between dynamic movements and static postures while focusing their attention and consciousness on the present body and excluding all other thoughts. This process has brought about physiological changes in the brain and enhanced the comprehensive communication between cognitive and emotional brain regions, thus verifying the hypothesis that sport can improve hot EF (Leshem et al., 2020). Therefore, physical sport is not only beneficial to physical health but also closely related to the development of cognition, emotion, and decision-making of hot EF.

Given the significant value of hot EF, researchers actively explored various intervention methods, with sport training and music training emerging as widely used approaches. Participation in music activities generated a musical advantage, which refers to a non-musical cognitive benefit acquired through formal musical training (Benítez-Barrera et al., 2022). Putkinen et al. (2015) explored the effects of formal music training and informal music group activities on children’s cognitive abilities. The findings revealed that music training not only enhanced children’s neural audio discrimination but also improved their attention control skills, exemplifying the positive effects of music engagement on cognitive development. A study randomly selected 36 children with abnormal emotional socialization development as research participants to implement music intervention, and they evaluated the children’s emotional and social development again 3 months later. After music intervention, the indicators of the children’s emotional socialization development in the implicit behavior domain had improved most obviously, and the dimensions of impulsiveness and anxiety in activities had also improved significantly, showing statistical differences with the pre-test results (Feiying et al., 2018). Musical training can also enhance neuroplasticity and structural brain development (Hyde et al., 2009; Luo et al., 2012). The study involved college students reflecting on their music training experiences and revealed the positive impact of music training on children’s emotional and cognitive processes. Music training has a positive impact on people’s affective decision making by enhancing emotional processing, improving cognitive abilities, cultivating attention and concentration, as well as promoting brain development and neural connections (Hou et al., 2017). At the same time, musical training can also lead to better emotional arousal and make the emotional state develop in a positive direction (Husain et al., 2002; Shaposhnikova et al., 2021).

Rhythmic-movement activities are generally defined as a type of activity that combines musical rhythm with physical movement (Miendlarzewska and Trost, 2014; Williams and Berthelsen, 2019; Hu et al., 2020; Laure and Habe, 2023). In China’s childhood education, rhythmic-movement activities usually exist as a kind of music teaching curriculum. In this activity, teachers guide children to accompany music, express music content through rhythmic movements, perceive the elements of music, and develop concentration, imagination and movement coordination. In order to attract the interest of children, preschool teachers usually provide rewards to be awarded at the end of the activity. Relevant studies have shown that whether gifts are presented in front of them will also affect children’s delayed gratification level (Mischel, 1974). And the speed and accuracy of decision making is also improved by the incentive of rewards (Geier et al., 2010; Padmanabhan et al., 2011). Therefore, our intervention activity integrated the reward element. Studies have shown that children can indeed perform rhythmic physical activity to music (Einarson and Trainor, 2016; Kragness and Trainor, 2018; Tuominen et al., 2020). And this activity aims to teach children to hear and to respond organically to the various rhythms of music; to make their bodies rhythmically coordinated and conscious through actually experiencing music in movement. The children are encouraged to move freely, vigorously, and naturally and to use their bodies as a whole (White, 2013). It includes dancing, clapping, tapping instruments, performing body movements, etc., which is widely used in early childhood education and loved by children. First, rhythmic-movement activities can improve children’s rhythmic ability (Venetsanou et al., 2014). A comparative study of music and rhythmic-movement activities found that rhythmic-movement activities not only improved children’s basic movement skills but also affected their rhythmic ability (Marinšek and Denac, 2020). Second, rhythmic-movement activities can promote children’s cognitive development (Williams and Berthelsen, 2019). Most researchers believe that the transfer effect of music training can promote the development of cognitive ability. Rhythmic-movement activity requires individuals to decode musical symbols and convert them into corresponding action commands, so as to gather attention during practice, which has a very positive impact on the development of spatial working memory, linguistic working memory, and reasoning ability of individuals. In addition, studies have also shown that music training can affect the gray matter volume of the temporal occipital lobe and insular cortex in the brain, and then promote the development of human cognition (Bergman Nutley et al., 2014). Researchist investigated the influence of rhythmic-movement activity on the cognition of children between the ages of three and four in a kindergarten in London in the United Kingdom. Music training can enhance the regional coordination of body movement, hearing, and perception (Bowmer et al., 2018). Third, rhythmic-movement activity has a certain influence on the development of emotions in hot EF. Rhythmic-movement activity can improve depression, stress, anxiety, and anger (Cevasco et al., 2005). And fourth, rhythmic-movement activity has an impact on children’s prosocial behavior and attitude (Wan and Zhu, 2021). Rhythm coordination in rhythmic-movement activity improves neural flexibility, and rhythmic-movement activity with cooperative behaviors can enhance prosocial behavior attitude and good mood because of the cooperative relationship with peers. When children participate in rhythmic-movement activity, they are easily mobilized by cheerful music. However, rhythmic-movement activity under the guidance of teachers requires children to concentrate on judging the rhythm to complete the combination of music and movements. Children must combine and regulate various cognitive abilities in their brains, so as to promote children’s control and recognition of emotions. At the same time, music activity is also a good means of emotional education for children, and its educational power is better than simply imparting knowledge (Zhou, 2015).

The process of rhythmic-movement activities typically includes warm-up; introduction; music appreciation; movement expression; music performance; and relaxation etc. (Matthews et al., 2016; Kim and Lee, 2023; Laure and Habe, 2023; Williams et al., 2023). A study designed a rhythm and movement intervention program to explore the effect of rhythm and movement intervention on improving the self-regulation skills of 3–4 year old children, which included the following steps: (i) warm-up involving body percussion; (ii) becoming familiar involving an adaptation of a familiar early childhood song; (iii) moving to the beat involving large grossmotor movements; (iv) playing to the beat involving simple rhythm sticks or castanets; (v) dancing to the beat involving slightly more complex gross motor movement patterns to activity three of the session and often involving visual motor skills and coordination such as mirroring the shape of rhythm sticks on the floor with bodies; and (vi) calming, which includes a series of movements and then stillness accompanied by relaxation music to support physiological entrainment to a calmer state, targeting embodied emotional regulation. After 8 weeks of intervention, the results of the study showed that rhythmic-movement activity had a significantly improved effect on children’s self-regulation (Williams et al., 2023). In another study, the researchers conducted a Montessori music movement activity to explore the development of rhythmic ability in 3–6 year old children. In the activity, children were deployed into two experimental groups and a control group. Intervention consisted of introducing 15–20 min of unstructured movement time, either accompanied by a piano or recording, three times a week for 4 months, whereas the control group carried on the usual Montessori program. Three tests for measuring rhythmic abilities were used: auditory discrimination of the rhythmic patterns, imitation of spoken rhythmic phrases, and determining the synchronization of movement with the rhythm of the music. It turned out that, Montessori music movement has a positive effect on the development of children’s rhythmic ability (Laure and Habe, 2023). Korean researchers developed an interpersonal relationship based music activity program for children from Grade 3 to Grade 6. This intervention activity included three stages: introduction music, development, and consolidate shared feelings. In the development stage, 10 interpersonal care techniques were applied in the theme area while performing activities, such as singing with Orff music, making ostinato rhythms, performing physical activities, playing musical instruments, and appreciating and playing musical instruments. The experiment showed that the intervention activity had significant effects on ego-resilience, peer relationships, happiness, interpersonal care awareness, physiological anxiety, and physiological stress (Kim and Lee, 2023). American researchers in rural southwestern Ohio conducted a study to understand how teachers experience and structure the role of rhythm in basic education. Through qualitative research methods, rhythm was incorporated into curriculum teaching by using music and rhythm, creating rhythmized learning environment, integrating art and music, and encouraging students to study independently, which effectively improves students’ learning effect and academic achievement (Matthews et al., 2016).

Therefore, this study designed and implemented an intervention measure targeting rhythmic-movement activities with rewards for children. The aim of this study was to determine the effects of this rhythmic-movement activities with rewards intervention on hot EF in children of 5 to 6 years old for 6 weeks. We hypothesized that the 6-week rhythmic-movement activities intervention would improve children’s hot EF. Specifically, the research hypothesis is as follows.

Hypothesis: rhythmic-movement activity with rewards intervention can significantly promote the development of hot EF in experimental group children.

## Materials and methods

### Participants

Participants were 62 Chinese 5- to 6-year-old children, who were from two classes of a preschool which was located in Yantai of China. To ensure the effect of regularizing the experimental response, two classes were randomly selected in the preschool, and children of one class were assigned to the control group (*M* = 5.77 years old, SD = 0.35 years old, 16 males and 16 females) and children of the other class were assigned to the experimental group (*M* = 5.83 years old, SD = 0.40 years old, 19 males and 11 females). Five children in the control group had experienced special music curriculums (dance and drum kit), as had three children in the experimental group (guzheng, piano, and dance). No parent of any child in either group had engaged in work related to music and art, and there was no statistical difference in age and gender between the experimental group and control group [gender *t*(62) = 1.050, *p* = 0.297]; [age *t*(62) = −0.652, *p* = 0.517]. All the children in the experimental group and control group were normal with no psychotic history, family genetic history, or serious physical diseases, and all had normal intelligence, hearing, and vision (in some cases corrected vision).

### Design and procedure

The rhythmic-movement activity with rewards intervention was conducted in a real-world setting, and interventions were carried out by the teachers in the class. To ensure the scientific nature of the experiment, the teacher did not know the purpose of the intervention, there by avoiding the subjective influence of the experimenters and verifying the operability of the framework. We employed a pre-post study design, and the participants of the two groups were assessed on the indexes of hot EF twice. Before the experiment, Informed Consent Forms were distributed to obtain parental consent and receive children’s basic information (name, gender, date of birth, participation of interest-oriented classes), and the parents of 62 children agreed to participate in the whole test. Having collected the information, pretests were completed within 2 weeks. All the children participated voluntarily in the tests and had the right to quit the experiment. At the start of experiment, the researchers explained the experimental requirements to the children to ensure that they understood the experimental tasks. After the pre-test, the experimental group conducted a 6-week intervention consisting of 18 lessons and each lesson consists of 6 sessions for 30–40 min; on the contrary, the control group received the routine teaching activities. The intervention process was as follows: warm-up; activity introduction and announcement of the rewards; music enjoyment; movement expression; music performance; relaxation and reward distribution. In the activities, all the children were encouraged to participate actively, show boldness, and participate in the challenges. Within 2 weeks after the intervention, the post-test of all the children was completed immediately. All the children in the experimental group and control group received the same two hot EF tests as the pre-test. The pre-test and post-test are the same person, and the intervention activities were organized and implemented by students majoring in pre-school education at Ludong University in China.

### Tests and materials

Two tasks, including the Gift Delay Task (GDT) and Children’s Gambling Task (ChGT), were used to test the two core components of hot EF. GD was measured using the GDT, and ADM ability was measured using the ChGT. The specific experimental operations were as follows.

In the current study, the tools for measuring gratification delay (GD) are: Gift Delay task (GDT) (Mischel et al., 1972; Zelazo et al., 2010) and Snack Delay Task (SDT) (Slot et al., 2017; Alesi et al., 2019), among which the former uses gifts as temptations, and the latter uses snacks as temptations. The basic procedures of the two are similar. A study have pointed out that the most commonly used instruments to assess hot EF in preschool children were the Gift Delay task (Montroy et al., 2019). In addition, we discussed the issue of temptation with the kindergarten where we intervened. Considering the safety of snacks, we finally chose the Gift Delay task. In this study, the self-GD paradigm proposed by Mischel was used for the GDT test to evaluate the developmental level of the children’s GD ability. First, before the experiment, the experimenter investigated the favorite toys of the children in the experimental group and control group and then showed screened pictures of the toys for the children to vote for their favorite ones. The statistical results were then used to select the toys that most of the children liked to determine the reward for GD. The preliminary preparation included a set of special tables and small stools for the children, a timer, an adult chair, and a pressing bell. The scoring method of the experiment was as follows: zero points for an immediate reward; one point for a delayed reward. The scoring standard for measuring delay maintenance ability was as follows: the standard time of this paradigm was 20 min, with every 4 min as a scoring segment, i.e., got one point in 0–4 min, got two points in 4.01–8 min, got three points in 8.01–12 min, got four points in 12.01–16 min and got five points in 116.01–20 min.

### Children’s gambling task

Tools for measuring affective decision making (ADM) include The Emotion Regulation Checklist (ERC) (Martins et al., 2020; Lucas-Molina et al., 2022) and Children’s Gambling Task (ChGT) (Andrews and Moussaumai, 2015; O'Toole et al., 2019; Beck et al., 2020). Firstly, the reason for choosing the ChGT to test affective decision-making is that the children’s Gambling Task simulates the interactive competition scene of children, which can better reflect children’s emotional adjustment ability in the real world. Secondly, children’s Gambling Task are repeatable and comparable. It can be repeated tests among different times, places and participants to assess the development and change of children’s emotional decision-making ability. Therefore, we chose the children’s game task as a measurement tool for emotional decision-making. The present study used the children’s version of the Iowa Gambling Task—also known as the ChGT—to test the children’s ADM ability of hot EF (Garon and Moore, 2004; Bunch et al., 2007; Hou et al., 2017). The main purpose of ADM is to make choices and decisions that are beneficial to individuals or to others (society). In the ChGT, children need to integrate rewards and punishment information from uncertain cards to win rewards to the maximum extent, and completing this experimental task requires high emotional input.

In the experiment, two decks of cards (20 cm × 30 cm) were used in this task: one deck of cards with 50 vertical stripes on the back, and the other deck of cards with 50 polka dots on the back. The test time was about 20 min. The vertical-stripe playing cards (advantage cards) always had a smiling face and occasionally a crying face. The polka-dot playing cards (disadvantage cards)always had two smiling faces and occasionally several crying faces (between four and six). The scoring standard for measuring ADM ability was as follows. The basic score of this test was 10 points. On this basis, the children randomly drew cards from the two stacks, winning or losing *X* points where *X* is the number of smiling faces minus the number of crying faces. After touching the cards 50 times, the test stopped and the children’s scores were counted. At this point, the children were asked four questions to test their understanding of the game cards. The first two questions focused on advantage cards, while the last two questions focused on disadvantage cards. The tester’s questions were as follows. Question i: Which set of cards is the best? If children can mention “vertical-stripe playing cards,” they will earn one point. Question ii: Why do you think this set of cards is the best? If children can say “these cards can earn me more smiles,” they will get one point. Question iii: Which set of cards is the worst? If children can mention “polka-dot playing cards,” they will earn one point. Question iv: Why do you think this set of cards is the worst? If children can say “these cards can make me lose many smiles,” they will earn one point. One point was given for each answer to a question, totaling four points. The total score of the test was the score of the cards plus the score of the questions.

### Intervention program

With the intervention, we aimed to develop the hot EF of children through rhythmic-movement activities with rewards. The intervention was done by the training teacher of rhythmic-movement activities with rewards, who was the main class teacher with 16 years of teaching experience of the experimental class and had a solid theoretical foundation in pre-school education. The training location was the activity room of the experimental group. The study selected 18 cheerful children’s songs, allowing children to perceive beats, rhythms, accents and melodies, to create movements along with the music for musical performance. In the activity, according to the content shared by children, teachers drew charts, showed the story plot, and explained the beats, rhythms, accents and melodies used in the music. Take rhythm as an example, reading “*ta*” in quarter rhythm with its chart of “*X*”; The musical instruments provided in the activity include African drums, sand hammers, double drum, carelets, etc. In addition, children were also provided with costumes for the performance.

Each activity in this study consists of 6 parts, and the activity design refers to the activity process of a rhythmic-movement activity with rewards intervention. (i) Warm-up means to attract the children’s attention and prepare the body. Teacher led children to sing while beating the body, such as “body syllable song”: “Touch your feet do do do do; Touch your knee re re re re…”; (ii) Activity introduction is to introduce the purpose and content of the activity and announce the rewards. The teacher presented the reward to the children and promised to receive the reward for their positive performance. Children can be rewarded when they completed the activity (the reward is non-test task reward); (iii) Music appreciation is that children appreciate music, imagine the story, and express the story with words and actions. The teacher draws a plot chart according to the content shared by the children; (iv) Movement expression means to enjoy music in sections, with the help of distinct character images and transformation of story plot, letting children use action to show music beat, strength, melody changes. For example, jumping shows the lively music, walking shows the music with slow rhythm; (v) Music performance is to perform a dance with different roles and music. According to the rhythm of music, the children can freely express their body movements, create body movements, gestures or use instruments combining audio-visual and movement. After getting familiar with the music plot and rhythm through the previous part, children looked at the chart and listened to the music to perform the song; (vi) Play soothing music to breathe deeply and calm children’s mood is the end of the activity part. Teacher led children to review the content of the activity, so that they can reflect on their own performance, in order to perform better in the following activities. Rewards would be presented upon completion of the activities.

Below are 18 intervention lesson songs (Table 1).

**Table 1:** 18 intervention lesson songs.

Week	Lesson	Songs
1	1	The Sun Bird Compliments Me 《太阳小鸟夸奖我》
	2	There Are Many Good Things in Kindergarten 《幼儿园里好事多》
	3	Role-playing House 《过家家》
2	4	Grandpa Makes Mooncakes for Me 《爷爷为我打月饼》
	5	Motherland, Motherland, We Love You 《祖国, 祖国, 我们爱你》
	6	Song of Reversal 《颠倒歌》
3	7	The Little Painter 《小小粉刷匠》
	8	Flying in the Blue Sky on the Small Airplane 《小飞机上蓝天》
	9	The Golden Peacock Jumps Gently 《金孔雀轻轻跳》
4	10	The Rustle of Spring Rain 《春雨沙沙》
	11	A Tiny Grain of Rice 《小小一粒米》
	12	The Window Opens, the Door Opens 《窗开开, 门开开》
5	13	Pulling the Hook 《拉拉钩》
	14	Teacher, Teacher, I Love You 《老师老师我爱你》
	15	The National Flag is Bright Red 《国家国旗红红的哩》
6	16	Taste Grapes 《尝葡萄》
	17	Cuckoo 《布谷鸟》
	18	Kneading Dough Man 《揉面团的人》

## Results

SPSS 27.0 was used to analyze the data. In this study, two groups (rhythmic-movement activity with rewards intervention training experimental group; control group) and two times (pre-experiment test; post-experiment test) were used to design the variance analysis, with the GD and ADM scores as the intra-subjectivity factors and the experimental and control classes as the inter-subjectivity factors. Two-way analysis of variance (ANOVA) was used to test the interaction between the experimental and control groups at the time points before and after the test.

Regarding the control of irrelevant factors, we first investigated the basic situation of the children (their participation in special curriculums, and their gender, age, and parents’ occupations), and there was no statistical difference. Second, before the experiment, a homogeneity test was carried out on the developmental level of GD and ADM ability of the children in the two groups. Regarding the intervention activities, the experimental group and control group had unified weekly and monthly teaching activities in the kindergarten, so the activities are similar. After school, the experimental group participated in 30–40 min of rhythmic-movement activity with rewards training three times a week for 6 weeks, while the control group received no intervention. The scores of the hot EF tasks before and after rhythmic-movement activity with rewards are given in Table 2.

**Table 2:** Task data of hot executive function (hot EF) in experimental group and control group.

	Experimental group (*n* = 30)		Control group (*n* = 32)	
	Pre-test (*M,* SD)	Post-test (*M,* SD)	Pre-test (*M,* SD)	Post-test (*M,* SD)
GDT	2.67 (0.96)	4.17 (1.62)	2.63 (1.01)	2.81 (1.03)
ChGT	3.60 (11.69)	13.83 (8.57)	0.50 (9.98)	0.75 (9.64)

### Statistical analysis of pre-test results of experimental and control groups

To better control the experimental variables, the test results for the two components of hot EF of the children in the experimental group and control group were analyzed using an independent-sample *T*-test to test whether the hot EF of the children in the experimental group and control group was at the same level. The results showed that there was no significant difference in the GD and ADM scores between the experimental group and control group.

There was no significant difference between the experimental group and control group in the pre-test scores of GD [*t*(62) = −0.167, *p* = 0.868], and there was no significant difference between the two groups in the pre-test scores of ADM ability [*t*(62) = −1.125, *p* = 0.265]. Therefore, the two ability levels of the two groups of children in the pre-test were equivalent, and intervention research could be carried out (Table 3).

**Table 3:** Pre-test data of experimental group and control group.

	Pre-test	N	M	SD	T	P
GDT	Control group	32	2.63	1.01	−0.167	0.868
	Experimental group	30	2.67	0.96		
ChGT	Control group	32	0.50	9.98	−1.125	0.265
	Experimental group	30	3.60	11.69		

### Effects of rhythmic-movement activity with rewards on children’s hot executive function, gratification delay, and affective decision-making ability

To study further the influence of rhythmic-movement activity with rewards on children’s GD and ADM, we conducted two-way bidirectional repeated-measurement ANOVA to analyze the interaction between the time points of GD and ADM (pre-test; post-test) and groups (rhythmic-movement activity with rewards experimental group; control group) (Table 4).

**Table 4:** Analysis of intervention effect of rhythmic-movement activity with rewards experimental group on hot EF.

Task	Source	df	MS	*F*	η^2^p
GDT	Time	1	20.976	25.077***	0.295
	Group	1	15.084	7.734	0.114
	Time × Group	1	13.337	15.944***	0.210
ChGT	Time	1	800.202	66.436***	0.525
	Group	1	2027.615	10.741	0.152
	Time × Group	1	771.615	64.063***	0.516

The GDT results showed that the interactions between time points and groups were significant [*F* = 13.337, *p* < 0.001, *η*^2^*p* = 0.210]. Simple effect analysis showed that there was significant difference between pre-test and post-test in the experimental group (*p* < 0.001) but no significant difference between pre-test and post-test in the control group (*p* = 0.415). Under the pre-test conditions, there was no significant difference between the experimental group and control group (*p* = 0.868). Under the post-test conditions, there was significant difference between the experimental group and control group (*p* < 0.001). This shows that rhythmic-movement activity with rewards can significantly promote the development of children’s GD ability.

The ChGT results showed that the interactions between time points and groups were significant [*F* = 64.063, *p* < 0.001, *η*^2^*p* = 0.516]. Simple effect analysis showed that there was significant difference between pre-test and post-test in experimental group (*p* < 0.001) but there was no significant difference between pre-test and post-test in control group (*p* = 0.774). Under the pre-test conditions, there was no significant difference between the experimental group and control group. Under the post-test conditions, there was significant difference between the experimental group and control group (*p* < 0.001). This shows that rhythmic-movement activity with rewards can significantly promote the development of childrens ADM ability.

## Discussion

### Analysis of effectiveness of rhythmic-movement activity with rewards intervention

Rhythmic-movement activity with rewards is a kind of music teaching course widely used in 3–6 year old children in China. It emphasizes that under the guidance of teachers, children express their understanding of music through different physical movements along with the rhythm of music, and integrate certain intellectual and social goals in the process. Generally, in the teaching process, teachers will jointly use rewards to stimulate children’s interest in participating in activities. If children successfully complete the activity, they will receive a small sticker representing honor. Study has shown that rhythm and physical movement play a role in improving hot EF (Vazou et al., 2020). Most of the previous studies explored the influence of rhythm or physical movement on the hot EF of children. Rhythmic-Movement activities can not only arouse children’s interest in participating in music activities, but also combine rhythm and physical movement. However, few studies have explored the relationship between rhythmic-movement activity with rewards and hot EF. Although some of the interventions in the past have achieved certain results, they are extremely challenging and difficult to implement when applied in standard educational settings. The rhythmic-movement activity with rewards in this study contains six basic parts, which makes the realization of rhythmic-movement intervention hot EF easier and more operable. The organizational form of rhythmic-movement activity with rewards is close to children’s life, and the intervention experiment was carried out in a kindergarten by means of games. A teacher with rich experience was selected as the organization teacher of intervention activities, which verifies the effectiveness and operability of the intervention framework in real kindergarten education, and lays a foundation for the better promotion and greater role of prosody activities in kindergartens.

Our study aims to explore the relationship between rhythmic-movement activity with rewards and hot EF in children. To examine these relationships, we designed rhythmic-movement activity with rewards to promote the development of hot EF in children, respectively measuring two sub-components of hot EF, including delayed gratification and affective decision-making ability in children. From the research results, the experimental results were consistent with the research hypothesis of this study. Rhythmic-movement activity with rewards can promote the development of children’s hot EF. To explain more clearly how an rhythmic-movement activity with rewards curriculum trains children’s hot EF, we discuss the influences of the two components separately.

### Effect of rhythmic-movement activity with rewards on ability to delay gratification

In this study, GD task was used to measure children’s delayed gratification ability before and after rhythmic-movement activity with rewards. The results showed that rhythmic-movement activity with rewards improved children’s performance in delay gratification, which is consistent with previous studies (Kenney, 2013; Cummings, 2014; Hennessy et al., 2019). Previous studies have analyzed the influence of rhythm-movement activity on delayed gratification from the perspective of cerebral cortex development and inhibitory control ability development (Holochwost et al., 2017; Jaschke et al., 2018). A study used functional magnetic resonance imaging confirmed that the brain regions associated with musical rhythm are located in the bilateral superior temporal cortex, auxiliary motor area, putamen, and cerebellum parts of the cerebral cortex, which are associated with the development of delayed gratification (Kasdan et al., 2022). In addition, some studies have found that listening to music can make people’s brain secrete a neurotransmitter called endorphins (Krout, 2007). Meanwhile this chemical substance can alleviate pain and anxiety in people, help them generate a sense of pleasure, and thus help them obtain satisfaction to a certain extent (Theorell and Theorell, 2014). Musical rhythm is also associated with the development of inhibitory control (Westby, 2012). Children adjusted their physical movements according to the perceived rhythm and perform music, which can promote the development of children’s inhibitory control and thus affect the development of delay gratification (Ramdass and Bembenutty, 2012; Traverso et al., 2015). Other studies have found that rhythm in music is also related to social aspect (Will, 2017). Young children and other children move together to the rhythm, creating a shared experience that also enhances interpersonal connection and satisfaction. Physical movements can affect delayed gratification through a variety of neural pathways and physiological mechanisms. Previous studies have confirmed that physical movements can activate motor neurons and other neural circuits and increase cerebrovascular flow in the prefrontal cortex, which is an important physiological basis for delayed gratification (Zayas et al., 2014; Graham and Brown, 2021; Douris et al., 2023). Physical movements are also closely related to neural functions such as cognitive processing, consciousness and memory, and may alter an individual’s perception and evaluation of satisfaction by activating specific brain structures and interconnected neural circuits (Johnson et al., 2013; Alleva et al., 2014). Therefore, physical movement can promote the development of delayed gratification. In addition, in rhythmic-movement activities, children perform music with physical movements to match their movements with the current music, inhibit the occurrence of incorrect movements, and thus achieve the effect of improving children’s inhibitory control ability. Rewards also affect the development of children’s ability to delay gratification (Miller and Karniol, 1976; Rapport et al., 1986). Rewards can be viewed as a positive feedback signal that increases the probability of a particular behavior occurring. Research has shown that the brain regions associated with reward overlap with those associated with delayed gratification, such as: dopaminergic (DAergic) neurons in the ventral tegmental area (Roesch and Olson, 2003). Rewards can also affect our mood and emotional state and studies have shown that how much children like the reward and whether the reward is presented to the child will affect the ability of children to delay gratification. Therefore, we carefully selected rewards for each lesson in the activity and displayed them in front of children’s eyes to enhance children’s motivation to get rewards, so as to control their behavior in the activity and improve the development of their ability to delay gratification.

### Effect of rhythmic-movement activity with rewards on affective decision-making ability

In this study, ChGT was used to measure children’s affective decision-making ability before and after rhythmic-movement activity with rewards. The results showed that rhythmic-movement activity with rewards could improve children’s affective decision-making ability. However, in previous studies, the effect of rhythmic-movement activity on affective decision-making ability is still controversial. One study explored the effect of rhythmic-movement activity on affective decision-making ability in young people and children, and collected the hot EF of the two age groups. The research results showed that there was no obvious correlation between rhythmic-movement activity and affective decision-making ability (Frischen et al., 2022). Another study compared the performance of three groups of participants (early, late, and no music training) on the Iowa Gambling Task (IGT) to examine whether rhythmic-movement activity was related to affective decision-making ability, and the results showed that early rhythmic-movement activity had a positive effect on affective decision-making ability (Hou et al., 2017), which was consistent with the results of our study. The rhythmic-movement activity intervention designed in this study also improved children’s performance in ChGT for the following reasons: First, the musical rhythm can promote the development of children’s affective decision-making ability. Previous studies have suggested that this effect may be caused by musical rhythm affecting children’s temporal, parietal and frontal cortex, as well as the thalamus and cerebellum. These brain regions were also involved in affective decision-making processes in children (Cardoso et al., 2014). At the same time, musical rhythm has been found to enhance the expression of dopamine D4 receptors (in the prefrontal cortex) (Cocker et al., 2014; Miendlarzewska and Trost, 2014), dopamine levels in the brain and associated genetic variants generally influence affective decision-making ability (He et al., 2010; Mapelli et al., 2014), improving children’s affective decision-making ability. In addition, research has shown that different rhythmic patterns can modulate emotional experience and decision-making processes (Thompson and Quinto, 2011; Zhou et al., 2022). Fast pacing is often associated with positive emotions and feelings of excitement. A fast pace can increase an individual’s level of emotional activation, promoting concentration and emotional arousal. This can lead to decisions that tend to be impulsive and seek immediate gratification. In contrast, a slow pace is often associated with relaxation and comfort. A slow pace can reduce an individual’s level of emotional activation, bringing a sense of calm and relaxation. This could lead to more careful, rational and long-term interest in decision-making (Cheng et al., 2009). Secondly, physical movement was also closely related to the development of affective decision-making ability (Gil-Arias et al., 2019). It has been found that physical movement enhanced people’s ability to organize and interpret sensory information, thereby developing more complex movement patterns and affecting their reasoning abilities as well as their ability to express and process emotions (McKay et al., 2019). Physical movements can also change an individual’s self-perception and level of confidence. This boost in self-confidence is likely to affect emotional decision-making, making it easier for individuals to take on challenges and try new things (Soytürk and Öztürk, 2019). In addition, the rewards given by teachers in this study may affect children’s affective decision-making ability to some extent. Rewards can trigger positive affective responses such as joy and excitement (Sander and Nummenmaa, 2021). These affective reactions will affect people’s perception and evaluation of things. Positive rewards make children more sensitive to the perception of the activities they participate in, thus affecting their affective decision-making (Brosch et al., 2010). Neurophysiological, functional neuroimaging and clinical neuropsychological studies have shown that the reward cortex is located in the orbitofrontal cortex and the anterior cingulate cortex, and this cortex is also a cortical region that can affect the development of affective decision-making ability (Diekhof et al., 2008). In addition, as an external incentive, rewards encourage children to be more inclined to adopt behaviors or choices that match rewards. When children expect a reward, they may be more motivated to try, learn, or complete a task. At the same time, the incentive of rewards can also improve the speed and accuracy of decision-making (Khodadadi et al., 2014). In this study, rewards given by teachers have an impact on children’s affective decision-making ability, which may also be related to teachers’ positive feedback (Schiebener and Brand, 2015). When children actively complete a rhythmic-movement activity with rewards, the teacher will give positive reward feedback. This kind of positive feedback can enhance children’s self-confidence and enthusiasm, and play a positive incentive role for their affective decision-making.

In conclusion, the development of delayed gratification and affective decision-making ability is a complex and multi-factorial process involving the interaction and integration of multiple factors, and our research on this aspect is still evolving.

### Limitations and future directions

Five major limitations of this study should be noted. First of all, the intervention activity contains three important elements (musical rhythm, physical movement and rewards). Although relevant contents are covered in the discussion section, we have not been able to identify the respective contributions of the 3 components in improving hot EF, which is an important research direction in the future. Second, there has been no discussion about the interaction of multivariate variables such as the content, organization, and duration of the rhythmic-movement activity. Third, there was no further study of the potential neural mechanism of children’s hot EF by using brain imaging and other technologies. Forth, there may be some uncontrollable variables in the quasi-experimental design of the experimental and control groups in the real educational environment of the kindergarten. Finally, there are defects in the selection and intervention of GDT, which makes the progress of the low-level group not obvious, and there was no continuous follow-up investigation on the development of hot EF. Nevertheless, the results of this study are promising. In future work, we can further determine the respective contributions of these three components in increasing hot EF, and we will extend the training time, explore which steps in the program can improve the intervention effect, find a more suitable intervention plan and principle of rhythmic-movement activity, continue to discuss the organization and content of rhythmic-movement activity in depth, and track and investigate the long-term influence of rhythmic-movement activity on children’s hot EF. When measuring the level of GD, we should choose various measuring tools to better observe the influence of intervention activities on children’s hot EF. If conditions permit, we can also use brain imaging technology to observe the regional changes of the cerebral cortex, and think about irrelevant factors in kindergarten education to control them.

## Conclusion

In this study, the effects of rhythmic-movement activity with rewards on the hot EF in children of 5 to 6 years old, were investigated systematically by using standard experimental paradigms. It was found that rhythmic-movement activity with rewards can improve children’s GD and ADM ability and provide a new method for early intervention in children’s hot EF, which has important theoretical guiding value and practical significance.

## Data availability statement

The original contributions presented in the study are included in the article/supplementary material, further inquiries can be directed to the corresponding author.

## Ethics statement

The studies involving humans were approved by College of Elementary Education, Ludong University, Yantai City, Shandong Province, China. The studies were conducted in accordance with the local legislation and institutional requirements. Written informed consent for participation in this study was provided by the participants’ legal guardians/next of kin.

## Author contributions

SW: Conceptualization, Supervision, Writing – review & editing, Writing – original draft. AY: Data curation, Formal analysis, Writing – original draft, Writing – review & editing. XW: Conceptualization, Supervision, Writing – review & editing, Writing – original draft. RQ: Conceptualization, Writing – review & editing YC: Investigation, Writing – review & editing. WB: Investigation, Writing – review & editing. BH: Investigation, Writing – review & editing. CW: Investigation, Writing – review & editing.

## References

[ref1] Alesi M. Pecoraro D. Pepi A. (2019). Executive functions in kindergarten children at risk for developmental coordination disorder. Eur. J. Spec. Needs Educ. 34, 285–296. doi: 10.1080/08856257.2018.1468635

[ref2] Alleva J. M. Martijn C. Jansen A. Nederkoorn C. (2014). Body language: affecting body satisfaction by describing the body in functionality terms. Psychol. Women Q. 38, 181–196. doi: 10.1177/0361684313507897

[ref3] Andrews G. Moussaumai J. (2015). Improving children’s affective decision making in the Children’s gambling task. J. Exp. Child Psychol. 139, 18–34. doi: 10.1016/j.jecp.2015.05.005, PMID: 26068276

[ref4] Antonini T. N. Becker S. P. Tamm L. Epstein J. N. (2015). Hot and cool executive functions in children with attention-deficit/hyperactivity disorder and comorbid oppositional defiant disorder. J. Int. Neuropsychol. Soc. 21, 584–595. doi: 10.1017/S1355617715000752, PMID: 26416095 PMC4589250

[ref5] Bandyopadhyay D. Pammi V. S. C. Srinivasan N. (2013). “Role of affect in decision making” in Decision Making - Neural and Behavioural Approaches, 202. 10.1016/B978-0-444-62604-2.00003-4 23317825

[ref6] Beck D. M. Eales L. Carlson S. M. (2020). Hot and cool executive function and body mass index in young children. Cogn. Dev. 54:100883. doi: 10.1016/j.cogdev.2020.100883

[ref7] Benítez-Barrera C. R. Skoe E. Huang J. Tharpe A. M. (2022). Evidence for a musician speech-perception-in-noise advantage in school-age children. J. Speech Lang. Hear. Res. 65, 3996–4008. doi: 10.1044/2022_jslhr-22-00134 36194893

[ref8] Bergman Nutley S. Darki F. Klingberg T. (2014). Music practice is associated with development of working memory during childhood and adolescence. Front. Hum. Neurosci. 7:926. doi: 10.3389/fnhum.2013.00926 24431997 PMC3882720

[ref9] Bowmer A. Mason K. Knight J. Welch G. (2018). Investigating the impact of a musical intervention on preschool children’s executive function. Front. Psychol. 9:2389. doi: 10.3389/fpsyg.2018.02389, PMID: 30618906 PMC6307457

[ref10] Bracha A. Brown D. J. (2012). Affective decision making: A theory of optimism bias. Games Econ. Behav. 75, 67–80. doi: 10.1016/j.geb.2011.11.004

[ref11] Brosch T. Pourtois G. Sander D. (2010). The perception and categorisation of emotional stimuli: A review. Cognit. Emot. 24, 377–400. doi: 10.1080/02699930902975754

[ref12] Budde H. Voelcker-Rehage C. Pietraßyk-Kendziorra S. Ribeiro P. Tidow G. (2008). Acute coordinative exercise improves attentional performance in adolescents. Neurosci. Lett. 441, 219–223. doi: 10.1016/j.neulet.2008.06.024, PMID: 18602754

[ref13] Bunch K. M. Andrews G. Halford G. S. (2007). Complexity effects on the children's gambling task. Cogn. Dev. 22, 376–383. doi: 10.1016/j.cogdev.2007.01.004

[ref14] Cardoso C. O. Branco L. D. Cotrena C. Kristensen C. H. Schneider Bakos D. D. Fonseca R. P. (2014). The impact of frontal and cerebellar lesions on decision making: evidence from the Iowa gambling task. Front. Neurosci. 8:61. doi: 10.3389/fnins.2014.00061, PMID: 24782697 PMC3986592

[ref15] Cevasco A. M. Kennedy R. Generally N. R. (2005). Comparison of movement-to-music, rhythm activities, and competitive games on depression, stress, anxiety, and anger of females in substance abuse rehabilitation. J. Music. Ther. 42, 64–80. doi: 10.1093/jmt/42.1.64, PMID: 15839734

[ref16] Chang Y. K. Tsai Y. J. Chen T. T. Hung T. M. (2013). The impacts of coordinative exercise on executive function in kindergarten children: an ERP study. Exp. Brain Res. 225, 187–196. doi: 10.1007/s00221-012-3360-9 23239198

[ref17] Charpentier C. J. De Neve J. E. Li X. Roiser J. P. Sharot T. (2016). Models of affective decision making: how do feelings predict choice? Psychol. Sci. 27, 763–775. doi: 10.1177/0956797616634654, PMID: 27071751 PMC4904352

[ref18] Cheng F. F. Wu C. S. Yen D. C. (2009). The effect of online store atmosphere on consumer's emotional responses–an experimental study of music and colour. Behav. Inform. Technol. 28, 323–334. doi: 10.1080/01449290701770574

[ref19] Cocker P. J. Le Foll B. Rogers R. D. Winstanley C. A. (2014). A selective role for dopamine D4 receptors in modulating reward expectancy in a rodent slot machine task. Biol. Psychiatry 75, 817–824. doi: 10.1016/j.biopsych.2013.08.026, PMID: 24094512

[ref20] Çökük K. Kozikoglu I. (2020). A correlational study on primary school Students' School readiness and adaptation problems. Int. Online J. Educ. Teach. 7, 523–535. Available at: http://iojet.org/index.php/IOJET/article/view/831

[ref21] Corcoran R. P. Cheung A. C. K. Kim E. Xie C. (2018). Effective universal school-based social and emotional learning programs for improving academic achievement: A systematic review and meta-analysis of 50 years of research. Educ. Res. Rev. 25, 56–72. doi: 10.1016/j.edurev.2017.12.001

[ref22] Crone E. A. Steinbeis N. (2017). Neural perspectives on cognitive control development during childhood and adolescence. Trends Cogn. Sci. 21, 205–215. doi: 10.1016/j.tics.2017.01.003, PMID: 28159355

[ref23] Cummings D. (2014). Using music as a tool for promoting self-regulation and conflict resolution skills in preschool children. Available at: http://hdl.handle.net/10211.3/153829.

[ref24] Diamond A. (2006). “The early development of executive functions” in Lifespan cognition: Mechanisms of change, vol. 210, 70–95.

[ref25] Diekhof E. K. Falkai P. Gruber O. (2008). Functional neuroimaging of reward processing and decision-making: a review of aberrant motivational and affective processing in addiction and mood disorders. Brain Res. Rev. 59, 164–184. doi: 10.1016/j.brainresrev.2008.07.004, PMID: 18675846

[ref26] Douris P. C. Cottone J. Cruz P. Frosos N. Marino C. Singamenggala L.. (2023). The effects of externally paced exercise on executive function and stress in college-aged students. J. Sci. Sport Exerc. 5, 149–155. doi: 10.1007/s42978-022-00173-1

[ref27] Einarson K. M. Trainor L. J. (2016). Hearing the beat: young children’s perceptual sensitivity to beat alignment varies according to metric structure. Music Percept. Interdiscip. J. 34, 56–70. doi: 10.1525/MP.2016.34.1.56

[ref28] Fatwikiningsih N. (2016). Rehabilitasi neuropsikologi dalam upaya memperbaiki defisit executive function (fungsi eksekutif) klien gangguan mental. J. An-Nafs Kajian Penelitian Psikologi 1, 320–335. doi: 10.33367/psi.v1i2.296

[ref29] Feiying W. Yufei N. Yong N. Jengjeng J. Peng H. (2018). Exploring the influence of music games on children's emotional socialization. Chinese J. Maternal Child Health 5, 34–36. doi: 10.19757/j.cnki.issn1674-7763.2018.05.008

[ref30] Frischen U. Schwarzer G. Degé F. (2022). Music training and executive functions in adults and children: what role do hot executive functions play? Z. Erzieh. 25, 551–578. doi: 10.1007/s11618-022-01103-1

[ref31] Garon N. Moore C. (2004). Complex decision-making in early childhood. Brain Cogn. 55, 158–170. doi: 10.1016/s0278-2626(03)00272-0 15134850

[ref32] Geier C. F. Terwilliger R. Teslovich T. Velanova K. Luna B. (2010). Immaturities in reward processing and its influence on inhibitory control in adolescence. Cereb. Cortex 20, 1613–1629. doi: 10.1093/cercor/bhp225, PMID: 19875675 PMC2882823

[ref33] Gil-Arias A. Garcia-Gonzalez L. Alvarez F. D. V. Gallego D. I. (2019). Developing sport expertise in youth sport: a decision training program in basketball. PeerJ 7:e7392. doi: 10.7717/peerj.7392, PMID: 31423354 PMC6697037

[ref34] Graham J. D. Brown D. M. (2021). “Understanding and interpreting the effects of prior cognitive exertion on self-regulation of sport and exercise performance” in Motivation and self-regulation in sport and exercise (Routledge), 113–133. Available at: https://www.taylorfrancis.com/chapters/edit/10.4324/9781003176695-9/understanding-interpreting-effects-prior-cognitive-exertion-self-regulation-sport-exercise-performance-jeffrey-graham-denver-brown

[ref35] He Q. Xue G. Chen C. Lu Z. Dong Q. Lei X.. (2010). Serotonin transporter gene-linked polymorphic region (5HTTLPR) influences decision making under ambiguity and risk in a large Chinese sample. Neuropharmacology 59, 518–526. doi: 10.1016/j.neuropharm.2010.07.008, PMID: 20659488 PMC2946467

[ref36] Hennessy S. L. Sachs M. E. Ilari B. Habibi A. (2019). Effects of music training on inhibitory control and associated neural networks in school-aged children: A longitudinal study. Front. Neurosci. 13:1080. doi: 10.3389/fnins.2019.01080, PMID: 31680820 PMC6805726

[ref37] Holochwost S. J. Propper C. B. Wolf D. P. Willoughby M. T. Fisher K. R. Kolacz J.. (2017). Music education, academic achievement, and executive functions. Psychol. Aesthet. Creat. Arts 11, 147–166. doi: 10.1037/aca0000112

[ref38] Hou J. He Q. Chen C. Dong Q. (2017). Early musical training contributes to decision-making ability. Psychomusicology 27, 75–80. doi: 10.1037/pmu0000174

[ref39] Hu X. Jiang G. P. Ji Z. Q. Pang B. Liu J. (2020). Effect of novel rhythmic physical activities on fundamental movement skills in 3-to 5-year-old children. Biomed. Res. Int. 2020, 1–10. doi: 10.1155/2020/8861379, PMID: 33426079 PMC7781705

[ref40] Husain G. Thompson W. F. Schellenberg E. G. (2002). Effects of musical tempo and mode on arousal, mood, and spatial abilities. Music. Percept. 20, 151–171. doi: 10.1525/mp.2002.20.2.151

[ref41] Hyde K. L. Lerch J. Norton A. Forgeard M. Winner E. Evans A. C.. (2009). Musical training shapes structural brain development. J. Neurosci. 29, 3019–3025. doi: 10.1523/JNEUROSCI.5118-08.2009, PMID: 19279238 PMC2996392

[ref42] Jaschke A. C. Honing H. Scherder E. J. (2018). Longitudinal analysis of music education on executive functions in primary school children. Front. Neurosci. 12:103. doi: 10.3389/fnins.2018.00103, PMID: 29541017 PMC5835523

[ref43] Johnson P. Fallon E. A. Harris B. S. Burton B. (2013). Body satisfaction is associated with Transtheoretical model constructs for physical activity behavior change. Body Image 10, 163–174. doi: 10.1016/j.bodyim.2012.12.002, PMID: 23339856

[ref44] Kasdan A. V. Burgess A. N. Pizzagalli F. Scartozzi A. Chern A. Kotz S. A.. (2022). Identifying a brain network for musical rhythm: A functional neuroimaging meta-analysis and systematic review. Neurosci. Biobehav. Rev. 136:104588. doi: 10.1016/j.neubiorev.2022.104588, PMID: 35259422 PMC9195154

[ref45] Kenney S. H. (2013). Moving music, mapping music: connecting children to the classics. General Music Today 26, 44–47. doi: 10.1177/1048371312474463

[ref46] Kerr A. Zelazo P. D. (2004). Development of “hot” executive function: the children’s gambling task. Brain Cogn. 55, 148–157. doi: 10.1016/s0278-2626(03)00275-6, PMID: 15134849

[ref47] Khodadadi A. Fakhari P. Busemeyer J. R. (2014). Learning to maximize reward rate: a model based on semi-Markov decision processes. Front. Neurosci. 8:101. doi: 10.3389/fnins.2014.00101 24904252 PMC4033239

[ref48] Kim S. H. Lee S. (2023). Effects of an Orff music activity intervention program on the Ego-resilience, peer relationships, happiness, interpersonal care awareness, anxiety, and stress of children from multicultural families in Republic of Korea[C]//healthcare. MDPI 11:2095. doi: 10.3390/healthcare11142095, PMID: 37510536 PMC10379974

[ref49] Kochanska G. Murray K. Jacques T. Y. Koenig A. L. Vandegeest K. A. (1996). Inhibitory control in young children and its role in emerging internalization. Child Dev. 67, 490–507. doi: 10.1111/j.1467-8624.1996.tb01747.x, PMID: 8625724

[ref50] Kragness H. E. Trainor L. J. (2018). Young children pause on phrase boundaries in self-paced music listening: the role of harmonic cues. Dev. Psychol. 54, 842–856. doi: 10.1037/dev0000405 29355358

[ref51] Krout R. E. (2007). Music listening to facilitate relaxation and promote wellness: integrated aspects of our neurophysiological responses to music. Arts Psychother. 34, 134–141. doi: 10.1016/j.aip.2006.11.001

[ref52] Laure M. Habe K. (2023). Stimulating the development of rhythmic abilities in preschool children in Montessori kindergartens with music-movement activities: A quasi-experimental study. Early Childhood Educ. J. 3, 1–12. doi: 10.1007/s10643-023-01459-x

[ref53] Lerner J. S. Li Y. Valdesolo P. Kassam K. S. (2015). Emotion and decision making. Annu. Rev. Psychol. 45, 133–155. doi: 10.1016/0001-6918(80)90026-8 25251484

[ref54] Leshem R. De Fano A. Ben-Soussan T. D. (2020). The implications of motor and cognitive inhibition for hot and cool executive functions: the case of quadrato motor training. Front. Psychol. 940:11. doi: 10.3389/fpsyg.2020.00940 PMC7250031 32508720

[ref55] Logue S. F. Gould T. J. (2014). The neural and genetic basis of executive function: attention, cognitive flexibility, and response inhibition. Pharmacol. Biochem. Behav. 123, 45–54. doi: 10.1016/j.pbb.2013.08.007, PMID: 23978501 PMC3933483

[ref56] Lucas-Molina B. Giménez-Dasí M. Quintanilla L. Gorriz-Plumed A. B. Giménez-García C. Sarmento-Henrique R. (2022). Spanish validation of the emotion regulation checklist (ERC) in preschool and elementary children: relationship with emotion knowledge. Soc. Dev. 31, 513–529. doi: 10.1111/sode.12585

[ref57] Luo C. Guo Z. W. Lai Y. X. Liao W. Liu Q. Kendrick K. M.. (2012). Musical training induces functional plasticity in perceptual and motor networks: insights from resting-state FMRI. PLoS One 7:e36568. doi: 10.1371/journal.pone.0036568, PMID: 22586478 PMC3346725

[ref58] Mandolesi L. Polverino A. Montuori S. Foti F. Ferraioli G. Sorrentino P.. (2018). Effects of physical exercise on cognitive functioning and wellbeing: biological and psychological benefits. Front. Psychol. 9:509. doi: 10.3389/fpsyg.2018.00509, PMID: 29755380 PMC5934999

[ref59] Mapelli D. Di Rosa E. Cavalletti M. Schiff S. Tamburin S. (2014). Decision and dopaminergic system: an ERPs study of Iowa gambling task in Parkinson’s disease. Front. Psychol. 5:684. doi: 10.3389/fpsyg.2014.00684 25071654 PMC4080179

[ref60] Marinšek M. Denac O. (2020). The effects of an integrated programme on developing fundamental movement skills and rhythmic abilities in early childhood. Early Childhood Educ. J. 48, 751–758. doi: 10.1007/s10643-020-01042-8

[ref61] Martin L. N. Delgado M. R. (2011). The influence of emotion regulation on decision-making under risk. J. Cogn. Neurosci. 23, 2569–2581. doi: 10.1162/jocn.2011.21618, PMID: 21254801 PMC3164848

[ref62] Martins E. C. Mărcuș O. Leal J. Visu-Petra L. (2020). Assessing hot and cool executive functions in preschoolers: affective flexibility predicts emotion regulation. Early Child Dev. Care 190, 1667–1681. doi: 10.1080/03004430.2018.1545765

[ref63] Matthews D. R. Ubbes V. A. Freysinger V. J. (2016). A qualitative investigation of early childhood teachers’ experiences of rhythm as pedagogy. J. Early Child. Res. 14, 3–17. doi: 10.1177/1476718X14523745

[ref64] McKay C. D. Cumming S. P. Blake T. (2019). Youth sport: friend or foe? Best Pract. Res. Clin. Rheumatol. 33, 141–157. doi: 10.1016/j.berh.2019.01.017 31431268

[ref65] Miendlarzewska E. A. Trost W. J. (2014). How musical training affects cognitive development: rhythm, reward and other modulating variables. Front. Neurosci. 7:279. doi: 10.3389/fnins.2013.00279, PMID: 24672420 PMC3957486

[ref66] Miller D. T. Karniol R. (1976). The role of rewards in externally and self-imposed delay of gratification. J. Pers. Soc. Psychol. 33, 594–600. doi: 10.1037/0022-3514.33.5.594

[ref67] Mischel W. (1974). “Processes in delay of gratification” in Advances in experimental social psychology, vol. 7 (Academic Press), 249–292. doi: 10.1016/S0065-2601(08)60039-8

[ref68] Mischel W. (2014). The marshmallow test: understanding self-control and how to master it. Random House 311:92.

[ref69] Mischel W. Ebbesen E. B. Raskoff Zeiss A. (1972). Cognitive and attentional mechanisms in delay of gratification. J. Pers. Soc. Psychol. 21, 204–218. doi: 10.1037/h0032198, PMID: 5010404

[ref70] Montroy J. J. Merz E. C. Williams J. M. Landry S. H. Johnson U. Y. Zucker T. A.. (2019). Hot and cool dimensionality of executive function: model invariance across age and maternal education in preschool children. Early Child. Res. Q. 49, 188–201. doi: 10.1016/j.ecresq.2019.06.011

[ref71] Moriguchi Y. Shinohara I. Yanaoka K. (2018). Neural correlates of delay of gratification choice in young children: near-infrared spectroscopy studies. Dev. Psychobiol. 60, 989–998. doi: 10.1002/dev.21791, PMID: 30368795

[ref72] O'Toole S. E. Tsermentseli S. Humayun S. Monks C. P. (2019). Cool and hot executive functions at 5 years old as predictors of physical and relational aggression between 5 and 6 years old. Int. J. Behav. Dev. 43, 157–165. doi: 10.1177/0165025418798498

[ref73] Padmanabhan A. Geier C. F. Ordaz S. J. Teslovich T. Luna B. (2011). Developmental changes in brain function underlying the influence of reward processing on inhibitory control. Dev. Cogn. Neurosci. 1, 517–529. doi: 10.1016/j.dcn.2011.06.004, PMID: 21966352 PMC3181104

[ref74] Prencipe A. Kesek A. Cohen J. Lamm C. Lewis M. D. Zelazo P. D. (2011). Development of hot and cool executive function during the transition to adolescence. J. Exp. Child Psychol. 108, 621–637. doi: 10.1016/j.jecp.2010.09.008, PMID: 21044790

[ref75] Putkinen V. Tervaniemi M. Saarikivi K. Huotilainen M. (2015). Promise of formal and informal musical activities in advancing neurocognitive development throughout childhood. Ann. N. Y. Acad. Sci. 1337, 153–162. doi: 10.1111/nyas.12656, PMID: 25773630

[ref76] Racz S. J. King K. M. Wu J. Witkiewitz K. McMahon R. J. the Conduct Problems Prevention Research Group (2013). The predictive utility of a brief kindergarten screening measure of child behavior problems. J. Consult. Clin. Psychol. 81, 588–599. doi: 10.1037/a0032366, PMID: 23544679 PMC3752994

[ref77] Ramdass D. Bembenutty H. (2012). Exploring self-regulatory behaviors during music practice among south Asian Indian American instrumental students. Int. J. Res. Rev. 59, 321–338. doi: 10.1177/0022429411414717

[ref78] Rapport M. D. Tucker S. B. DuPaul G. J. Merlo M. Stoner G. (1986). Hyperactivity and frustration: the influence of control over and size of rewards in delaying gratification. J. Abnorm. Child Psychol. 14, 191–204. doi: 10.1007/bf00915440, PMID: 3722617

[ref79] Roesch M. R. Olson C. R. (2003). Impact of expected reward on neuronal activity in prefrontal cortex, frontal and supplementary eye fields and premotor cortex[J]. J. Neurophysiol. 90, 1766–1789. doi: 10.1152/jn.00019.2003, PMID: 12801905

[ref80] Rueda M. R. Paz-Alonzo P. M. (2013). Executive function and emotional development contexts 1, 2. Available at: https://www.child-encyclopedia.com/pdf/complet/executive-functions

[ref81] Rueda M. R. Posner M. I. Rothbart M. K. (2016). “The development of executive attention: contributions to the emergence of self-regulation” in Measurement of executive function in early childhood, vol. 28 (Psychology Press), 573–594. 10.1207/s15326942dn2802_2 16144428

[ref82] Sander D. Nummenmaa L. (2021). Reward and emotion: an affective neuroscience approach. Curr. Opin. Behav. Sci. 39, 161–167. doi: 10.1016/j.cobeha.2021.03.016

[ref83] Schiebener J. Brand M. (2015). Decision making under objective risk conditions–a review of cognitive and emotional correlates, strategies, feedback processing, and external influences. Neuropsychol. Rev. 25, 171–198. doi: 10.1007/s11065-015-9285-x, PMID: 25894847

[ref84] Schlam T. R. Wilson N. L. Shoda Y. Mischel W. Ayduk O. (2013). Preschoolers' delay of gratification predicts their body mass 30 years later. J. Pediatr. 162, 90–93. doi: 10.1016/j.jpeds.2012.06.049, PMID: 22906511 PMC3504645

[ref85] Shakehnia F. Amiri S. Ghamarani A. (2021). The comparison of cool and hot executive functions profiles inchildren with ADHD symptoms and normal children. Asian J. Psychiatr. 55:e102483:102483. doi: 10.1016/j.ajp.2020.102483 33271479

[ref86] Shaposhnikova I. I. Korsun S. M. Arefieva L. P. Kostikova O. V. Serhiienko V. M. Korol S. A.. (2021). Analysis of students' somatic health and emotional state during sports games classes. Wiad Lek 74, 608–612. doi: 10.36740/WLek202103208 33843621

[ref87] Simpson A. Riggs K. J. (2007). Under what conditions do young children have difficulty inhibiting manual actions? Dev. Psychol. 43, 417–428. doi: 10.1037/0012-1649.43.2.417, PMID: 17352549

[ref88] Slot P. L. Mulder H. Verhagen J. Leseman P. P. (2017). Preschoolers' cognitive and emotional self-regulation in pretend play: relations with executive functions and quality of play. Infant Child Dev. 26:e2038. doi: 10.1002/icd.2038

[ref89] Sohrabi T. (2019). Physical education games and social skills: an investigation with Iranian primary school girls. Issues Educ. Res. 29, 1313–1329. doi: 10.3316/informit.721958808721142

[ref90] Soytürk M. Öztürk Ö. T. (2019). A comparison of ninth-grade Students' states of self-esteem and decision-making with respect to their levels of physical activity. Int. Educ. Stud. 12, 9–18. doi: 10.5539/ies.v12n4p9

[ref91] Theorell T. Theorell T. (2014). “Music for body and soul: physiological effects of listening to music” in Psychological Health Effects of Musical Experiences: Theories, Studies and Reflections in Music Health Science, 33–47.

[ref92] Thompson W. F. Quinto L. (2011). “Music and emotion: psychological considerations” in The aesthetic mind: Philosophy and psychology, 357–375.

[ref93] Traverso L. Viterbori P. Usai M. C. (2015). Improving executive function in childhood: evaluation of a training intervention for 5-year-old children. Front. Psychol. 6:525. doi: 10.3389/fpsyg.2015.00525, PMID: 25983706 PMC4415324

[ref94] Tuominen P. P. Raitanen J. Rinne M. (2020). The test–retest repeatability of a rhythm coordination test procedure in 4-to 6-year-old children: A pilot study. Music Sci. 3:2059204320962281. doi: 10.1177/2059204320962281

[ref95] Vazou S. Klesel B. Lakes K. D. Smiley A. (2020). Rhythmic physical activity intervention: exploring feasibility and effectiveness in improving motor and executive function skills in children. Front. Psychol. 11:556249. doi: 10.3389/fpsyg.2020.556249, PMID: 33071879 PMC7530936

[ref96] Veer I. M. Luyten H. Mulder H. van Tuijl C. Sleegers P. J. (2017). Selective attention relates to the development of executive functions in 2, 5-to 3-year-olds: A longitudinal study. Early Child. Res. Q. 41, 84–94. doi: 10.1016/j.ecresq.2017.06.005

[ref97] Venetsanou F. Donti O. Koutsouba M. (2014). The effect of a music/movement program on preschooler’s motor rhythmic ability. Eur. Psychomotricity J. 6, 60–73. Available at: https://psychomotor.gr/wp-content/uploads/2018/02/8.VENETSANOU_60_72.pdf

[ref98] Volkow N. D. Fowler J. S. Wang G. J. Telang F. Logan J. Jayne M.. (2010). Cognitive control of drug craving inhibits brain reward regions in cocaine abusers. NeuroImage 49, 2536–2543. doi: 10.1016/j.neuroimage.2009.10.088, PMID: 19913102 PMC2818484

[ref99] Wan Y. Zhu L. (2021). Effects of rhythmic turn-taking coordination on five-year-old children’s prosocial behaviors. Dev. Psychol. 57, 1787–1795. doi: 10.1037/dev0001244, PMID: 34914445

[ref100] Westby C. (2012). Self-regulation and delaying gratification. Word Mouth 23, 1–5. doi: 10.1177/1048395012440418

[ref101] White E. (2013). Outline of objectives and materials for rhythmic activities for children. J. Health Phys. Educ. 6, 29–54. doi: 10.1080/23267240.1935.10626307

[ref102] Will U. (2017). “Cultural factors in responses to rhythmic stimuli” in Rhythmic stimulation procedures in neuromodulation (Academic Press), 279–306.

[ref103] Williams K. E. Bentley L. A. Savage S. Eager R. Nielson C. (2023). Rhythm and movement delivered by teachers supports self-regulation skills of preschool-aged children in disadvantaged communities: A clustered RCT. Early Child. Res. Q. 65, 115–128. doi: 10.1016/j.ecresq.2023.05.008

[ref104] Williams K. E. Berthelsen D. (2019). Implementation of a rhythm and movement intervention to support self-regulation skills of preschool-aged children in disadvantaged communities. Psychol. Music 47, 800–820. doi: 10.1177/0305735619861433

[ref105] Yanaoka K. Michaelson L. E. Guild R. M. Dostart G. Yonehiro J. Saito S.. (2022). Cultures crossing: the power of habit in delaying gratification. Psychol. Sci. 33, 1172–1181. doi: 10.1177/09567976221074650, PMID: 35749259 PMC9437728

[ref106] Yu J. Kam C. M. Lee T. M. (2016). Better working memory and motor inhibition in children who delayed gratification. Front. Psychol. 7:1098. doi: 10.3389/fpsyg.2016.01098, PMID: 27493638 PMC4955289

[ref107] Zayas V. Mischel W. Pandey G. (2014). “Mind and brain in delay of gratification” in The Neuroscience of Risky Decision Making, 145–176.

[ref108] Zelazo P. D. Carlson S. M. (2012). Hot and cool executive function in childhood and adolescence: development and plasticity. Child Dev. Perspect. 6, 354–360. doi: 10.1111/j.1750-8606.2012.00246.x

[ref109] Zelazo P. D. Cunningham W. A. (2007). “Executive function: mechanisms underlying emotion regulation” in Handbook of emotion regulation. ed. Gross J. J., vol. 3 (The Guilford Press), 115–117.

[ref110] Zelazo P. D. Müeller U. (2002). “Executive function in typical and atypical development” in Handbook of childhood cognitive developmentt.Oxford. ed. Goswami U. (Blackwell), 445–469.

[ref111] Zelazo P. D. Qu L. Kesek A. C. (2010). “Hot executive function: emotion and the development of cognitive control” in Child Development at the Intersection of Emotion and Cognition, 97–111.

[ref112] Zeng J. (2010). A Review of Research on Children’s Gambling Tasks. Campus Psychol. 8, 113–116. doi: 10.3969/j.issn.1673-1662.2010.02.017

[ref113] Zhou J. (2015). The value of music in children’s enlightenment education. Open J. Soc. Sci. 3, 200–206. doi: 10.4236/jss.2015.312023

[ref114] Zhou L. Yang Y. Li S. (2022). Music-induced emotions influence intertemporal decision making. Cognit. Emot. 36, 211–229. doi: 10.1080/02699931.2021.1995331, PMID: 34702138

